# Bipedal locomotion in zoo apes: Revisiting the hylobatian model for bipedal origins

**DOI:** 10.1017/ehs.2022.9

**Published:** 2022-03-14

**Authors:** Kyle H. Rosen, Caroline E. Jones, Jeremy M. DeSilva

**Affiliations:** 1 Department of Anthropology, Dartmouth College, 6047 Silsby Hall, Hanover, NH, USA; 2 Department of Psychology, University of Georgia, 125 Baldwin Street, Athens, GA, USA

**Keywords:** Bipedalism, hominin, gibbon, hominid, hominoid

## Abstract

Bipedal locomotion is a hallmark of being human. Yet the body form from which bipedalism evolved remains unclear. Specifically, the positional behaviour (i.e. orthograde vs. pronograde) and the length of the lumbar spine (i.e. long and mobile vs. short and stiff) of the last common ancestor (LCA) of the African great apes and humans require further investigation. While fossil evidence would be the most conclusive, the paucity of hominid fossils from 5–10 million years ago makes this field of research challenging. In their absence, extant primate anatomy and behaviour may offer some insight into the ancestral body form from which bipedalism could most easily evolve. Here, we quantify the frequency of bipedalism in a large sample (*N =* 496) of zoo-housed hominoids and cercopithecines. Our results show that while each studied species of ape and monkey can move bipedally, hylobatids are significantly more bipedal and engage in bipedal locomotion more frequently and for greater distances than any other primate sampled. These data support hypotheses of an orthograde, long-backed and arboreal LCA, which is consistent with hominoid fossils from the middle-to-late Miocene. If true, knuckle-walking evolved in parallel in *Pan* and *Gorilla*, and the human body form, particularly the long lower back and orthograde posture, is conserved.

## Graphical abstract

**Figure:**
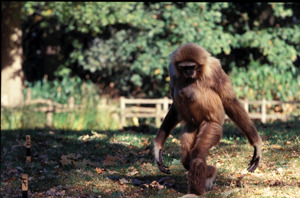

**Social media summary:** Gibbons and siamangs – the so-called ‘lesser apes’ – move on two legs a lot, and that might give us some clues into the origins of bipedal locomotion in human ancestors.

## Introduction

**Figure 1.**
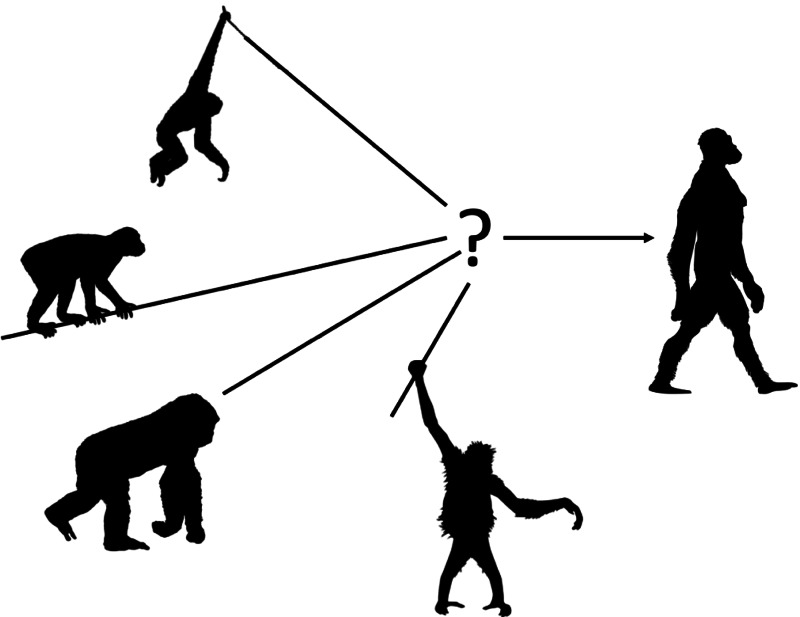
Evolution of bipedalism. The body form from which bipedalism evolved remains unknown. Scholars have proposed models based on (counterclockwise from top) brachiating hylobatids, pronograde monkeys, knuckle-walking African apes or quadrumanous orangutans. These models have deep roots in the anthropological literature and continue to be debated today. Figure based on Richmond et al. (2001), redrawn using PhyloPics Creative Commons Attribution-ShareAlike 3.0 Unported licence (https://creativecommons.org/licenses/by/3.0/), courtesy of Gareth Monger, T. Michael Keesey and Nobu Tamura.

Early twentieth-century physical anthropologists and anatomists had difficulty identifying the ape body plan from which the earliest human ancestors evolved. Keith (1923) identified homologous characters in living hominoids to posit an upright, orthograde ancestor that passed through a large-bodied, ‘Troglodytian’ phase. Gregory (1928) also envisioned an orthograde ancestor but hypothesised that bipedalism evolved from a gibbon-like brachiator as was suggested later by Avis (1962). Morton (1924) imagined a more generalised, small-bodied, quadrumanous ape capable of arboreal bipedal locomotion, although he also wrote of humans passing through a terrestrial ‘gorilloid’ phase. Straus (1949) diverged from this line of thinking and instead hypothesised that the earliest human ancestors descended from a primate that was more cercopithecoid-like – an above-branch, pronograde, arboreal quadruped (Figure 1).

The molecular revolution provided much needed clarity of our place in the primate family tree. Given that humans were more closely related to the African apes than any other primate (Sarich & Wilson, 1967), the knuckle-walking hypothesis became the most parsimonious (Gebo, 1996; Richmond, Begun, & Strait, 2001; Washburn, 1967) and the familiar March of Progress – in which a knuckle-walking ape steadily evolves into an upright human – became culturally (Howell, 1965) and academically (Wrangham & Pilbeam, 2002) entrenched.

However, despite a century of fossil discoveries and even clearer genetic evidence of a *Homo*–*Pan* clade (e.g. Chen & Li, 2001), our discipline appears to be no closer to identifying the body form from which bipedalism evolved than we were nearly a century ago (Figure 1). Some scholars have identified anatomical and behavioural inconsistencies with a knuckle-walking ancestor (Kivell & Schmitt, 2009; Tuttle, 1969), leading to the endorsement of a climbing, arboreal precursor to bipedalism (Crompton, Vereecke, & Thorpe, 2008; Fleagle et al., 1981; Tuttle, 1981). Data collected on wild orangutans further supports the hypothesis that bipedalism may have its roots in a clambering ape moving with hand-assisted bipedal locomotion in an arboreal environment (Thorpe, Holder, & Crompton, 2007). However, a more recent study of the 4.4 Ma *Ardipithecus ramidus* partial skeleton has resurrected Straus's (1949) cercopithecoid model in which the first hominins diverged from a pronograde, above-branch, arboreal quadruped (Lovejoy, Suwa, Simpson, Matternes, & White, 2009; White, Lovejoy, Asfaw, Carlson, & Suwa, 2015). Still, many scholars continue to support the most parsimonious hypothesis that bipedalism evolved from a knuckle-walking ancestor (e.g. Pilbeam & Lieberman, 2017; Prang et al., 2021). Whether using comparative anatomy, the fossil record or modern primate behaviour, our colleagues have presented reasonable, even convincing, evidence for their differing last common ancestor (LCA) reconstructions. Yet they cannot all be correct. One of these models (or another not yet proposed) is accurate, and the others will be scientifically refuted in time. The fact that our discipline has not reached that point evinces the need for more data.

A related argument has unfolded regarding the number of lumbar vertebrae present in the last common hominid ancestor. Humans possess an average of five lumbar vertebrae while modern great apes (chimpanzees, bonobos, gorillas and orangutans) have short, stiff lumbar regions with an average of three to four lumbar vertebrae (Williams, Gómez-Olivencia, & Pilbeam, 2019). The hylobatids are more human-like, with longer lumbar regions of four to six vertebrae, and cercopithecoids have the longest lumbar regions, with six or more vertebral elements (Williams et al., 2019). The polarity of vertebral number remains contentious, however. Some scholars favour an African ape-like, short-backed model for the common ancestor (Pilbeam, 2004; Williams, 2012; Williams, Middleton, Villamil, & Shattuck, 2016; Williams, Gómez-Olivencia, & Pilbeam, 2019), whereas others envision a long-backed ancestor (Böhme et al., 2019; Lovejoy et al., 2009; Lovejoy & McCollum, 2010; Machnicki & Reno, 2020; McCollum, Rosenman, Suwa, Meindl, & Lovejoy, 2010; Ward, Hammond, Plavcan, & Begun, 2019), although not all of these authors agree on whether the lumbar region would be human and gibbon-like (approximately five lumbar vertebrae) or more cercopithecoid-like (six or more lumbar vertebrae). Furthermore, a long-backed model would necessitate the parallel evolution of a short, stiff lumbar spine in orangutans, gorillas and the genus *Pan*.

Each of the different models for the body form of the African ape and human LCA is informed to various degrees by extant primate models. The terrestrial knuckle-walker hypothesis draws upon evidence from chimpanzees, bonobos and gorillas; the clambering arboreal bipedal hypothesis is based, in part, on evidence gathered from orangutans; the above-branch pronograde quadrupedal hypothesis draws upon skeletal evidence from extant cercopithecoids; and the hylobatian ancestor is informed by studies of gibbons and siamangs.

Fortunately, the pattern of positional behaviour and back anatomy in these primates lends itself to an examination of these differing hypotheses. If a short-backed, orthograde, knuckle-walking ape had a body most predisposed to bipedal locomotion, then chimpanzees, bonobos and gorillas might be expected to exhibit bipedalism most often. If, instead, the LCA was a clambering arboreal ape, orangutans might be the most frequent bipeds. If the last common ancestor was a long-backed, pronograde ape, then baboons and mandrills might be expected to exhibit the most frequent bipedal locomotion. The only animals in our sample with a combination of an orthograde body posture and a long lumbar region of the spine are hylobatids and, thus, high frequencies of bipedal locomotion in these lesser apes would align with aspects of the hylobatian model.

Here, we investigate the frequency of bipedalism in a large sample of zoo-housed primates. We posit that collecting these data on captive primates is preferable to wild observations for addressing our particular question given the different forest structures of the African and Asian rainforests, and in this way, zoo data may serve as a ‘control’ for ecological differences and allow us to focus specifically on anatomical predispositions for bipedal locomotion.

## Materials and methods

### Participants

To investigate which body form is most conducive to bipedal locomotion, we surveyed primate caretakers from 46 institutions with accreditation from the Association of Zoos and Aquariums (AZA) to quantify the frequency of bipedalism in a large sample (*N =* 496) of captive hominoids and cercopithecines (see Table 1). Specifically, we examined bonobos (*Pan paniscus*; *n =* 37), chimpanzees (*Pan troglodytes*; *n =* 82), gorillas (*Gorilla gorilla*; *n =* 115), orangutans (*Pongo* sp. *n =* 120), hylobatids (*Hylobates* sp. and *Symphalangus syndactylus*; *n =* 93), and cercopithecines (*Papio* sp. and *Mandrillus sphinx*; *n =* 49). We defined bipedal locomotion in our survey as movement of any distance on two legs. Postural bipedalism was not included.

**Table 1.** Sample demographic characteristics

Species	Age	Sex
Bonobo	Adult: 27 (73%) Juvenile: 10 (27%)	Female: 19 (51.4%) Male: 18 (48.6%)
Chimpanzee	Adult: 70 (85.4%) Juvenile: 11 (13.4%) Infant: 1 (1.2%)	Female: 45 (54.9%) Male: 37 (45.1%)
Gorilla	Adult: 90 (78.2%) Juvenile: 24 (20.9%) Infant: 1 (0.9%)	Female: 63 (54.8%) Male: 52 (45.2%)
Orangutan	Adult: 84 (70%) Juvenile: 27 (22.5%) Infant: 9 (7.5%)	Female: 65 (54.2%) Male: 55 (45.8%)
Hylobatid	Adult: 77 (82.8%) Juvenile: 13 (14%) Infant: 3 (3.2%)	Female: 44 (47.3%) Male: 47 (50.5%) Unknown: 2 (2.2%)
Cercopithecine	Adult: 42 (85.7%) Juvenile: 7 (14.3%)	Female: 28 (57.1%) Male: 21 (42.9%)

### Procedures

Requests for survey completion were sent to primate caretakers of AZA-accredited institutions that were also members of the Ape and Baboon Species Survival Plans. Surveys were developed and administered through the Qualtrics platform. The survey included six introductory/general questions and up to seven specific questions for each reported primate (see supplementary survey). Three key survey questions were used to assess the frequency of bipedalism in each primate (see Table 2): Question 1 assessed the occurrence of bipedalism using a multiple choice with multiple answer option format; Question 2 provided an open-ended response format to identify the average number of daily bipedal bouts in each primate; and Question 3 examined the average number of steps per bipedal bout in a multiple choice with single answer option format. If a respondent selected ‘No bipedal locomotion was observed in any of these timeframes’ in Question 1 for a particular primate, the survey terminated, and the respondent was prompted to begin a new set of responses for the next primate in their care. Accordingly, all responses for Questions 2 and 3 consisted of primates who were reported in Question 1 as bipedal.

**Table 2.** Survey questions assessing the frequency of bipedalism

Question	Response option(s)	Response type
Please select all that apply: I have observed bipedal locomotion in this primate at any point during …	□ This past month □ This past week □ Today □ No bipedal locomotion was observed in any of these timeframes	Multiple choice with multiple answer option
How many times did this primate exhibit this behaviour? Please give us your best numerical estimate if you do not recall the exact number of times. Please also indicate the appropriate timeframe (e.g. ‘X times per day/week/month’).	Open-ended response	Open-ended response
On average, how many steps did this primate take in each instance?	□ 1–3 □ 3–5 □ 5–7 □ 7–9 □ 10+	Multiple choice requiring single response

To identify the percentage of each species that has ever been observed to move bipedally, responses from Question 1 were dichotomised into two categories: (a) ‘Bipedal locomotion observed’ (including responses ‘This past month’, ‘This past week’ and ‘Today’); and (b) ‘No bipedal locomotion observed’ (including the response ‘No bipedal locomotion was observed in any of these timeframes’). Question 1 responses were not analysed across the original four response options because Question 2 provided the same information as Question 1 with additional quantitative benefits such as the specific number of bipedal bouts within an identified timeframe. Question 3 was asked in a multiple-choice format given the anticipated likelihood that an open-ended response option would yield responses spanning different ranges, thereby complicating comparisons across primates.

Additional information about the circumstances prompting primate bipedal locomotion was also collected in the survey and is the subject of ongoing analysis. Chi square analysis and paired-sample *t*-tests analyses were conducted using IBM SPSS Statistics (version 26).

## Results

**Figure 2.**
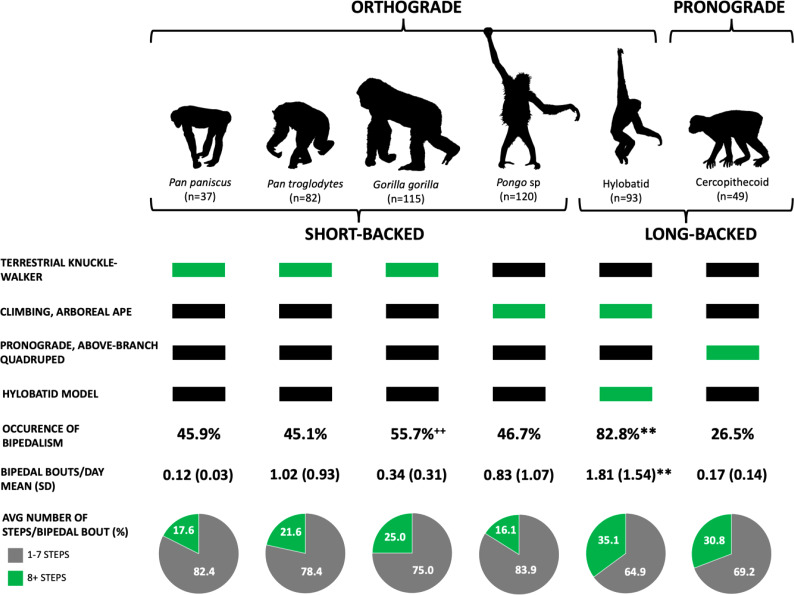
Bipedal behaviour in extant primates. Extant apes have an orthograde body plan, whereas monkeys are pronograde. Differences also exist in the number of lumbar vertebrae, with the great apes possessing three to four lower back vertebrae (short-backed) and hylobatids and cercopithecines having five to seven (long-backed). Humans and fossil hominins also have five lumbar vertebrae. We assess the different models for bipedal origins (listed along the left) and, if supported, which living apes are expected to exhibit the highest frequency of bipedalism (green rectangles). In our sample, 82.8% of the hylobatids (*n =* 77/93) moved bipedally, a value significantly higher than that for any other taxon. Furthermore, of the animals moving bipedally, hylobatids did it most frequently: 1.81 times per day. In contrast, a bipedal gorilla exhibits this behaviour, on average, once every three days. The long-backed primates – hylobatids and cercopithecines – also take the most steps per bipedal bout. ** *p* < 0.01; hylobatids were significantly more likely to be bipedal and had significantly more bipedal bouts per day compared with all other species. **^++^** *p* < 0.01; gorillas were significantly more likely to be bipedal compared with cercopithecines.

We find that species significantly differ by the occurrence of bipedalism (*χ*^2^ = 51.99, d.f.(5), *p* < 0.001; Figure 2). More specifically, *post hoc* analyses reveal significant differences in the occurrence of bipedalism between hylobatids and all other species, including bonobos (*p =* 0.001), chimpanzees (*p* < 0.001), gorillas (*p =* 0.001), orangutans (*p* < 0.001) and cercopithecines (*p* < 0.001). Additional significant differences were found between gorillas and cercopithecines (*p =* 0.005).

The results also show significant differences between species in the average number of daily bipedal bouts (*F* = 13.99, d.f.(5), *p* < 0.001). *Post hoc* analyses indicate that, among primates reported to move bipedally, significant differences exist between hylobatids and all other species, including bonobos (*p* < 0.001), chimpanzees (*p =* 0.008), gorillas (*p* < 0.001), orangutans (*p* < 0.001) and cercopithecines (*p* < 0.001). Species did not significantly differ relative to the number of steps per bipedal bout (*χ*^2^=7.39, d.f.(5), *p =* 0.193) although here, as well, hylobatids achieved the highest value (Figure 2).

## Discussion

The current findings demonstrate that hylobatids are both more likely to move bipedally and to do so more frequently than any other ape or monkey in a captive environment. While it has been suggested that bipedality in hylobatids is largely driven by their excessively long forelimbs (Lovejoy & McCollum, 2010), captive hylobatids have been documented to employ various terrestrial gaits, including quadrupedal and tripedal locomotion (Vereecke, D'Août, & Aerts, 2006). In fact, Vereecke et al. (2006) report frequent terrestrial quadrupedal locomotion in a population of captive gibbons unhindered by their elongated forelimbs. We thus interpret our findings as an indication that additional morphological features of the hylobatid body form, aside from their limb proportions, predispose them to walk bipedally more frequently than other primates – specifically, a long lumbar spine and orthograde positional behaviour. A limitation of this study is that we did not include any atelines, a family of primates that also possesses this combination of long lumbar spine and frequent orthograde positional behaviour.

To be sure, evolutionary trajectories do not always follow the path of least resistance. All of the primate taxa in our study practise some degree of bipedal locomotion, providing the behavioural and anatomical raw material for natural selection to favour this form of locomotion in early hominins no matter the body form of the LCA. In other words, if the strength of selection was sufficiently high, bipedal behaviour could have evolved from any of the models investigated in this study. Therefore, we cannot refute any of the hypotheses generated from modern models in a Popperian sense.

**Figure 3.**
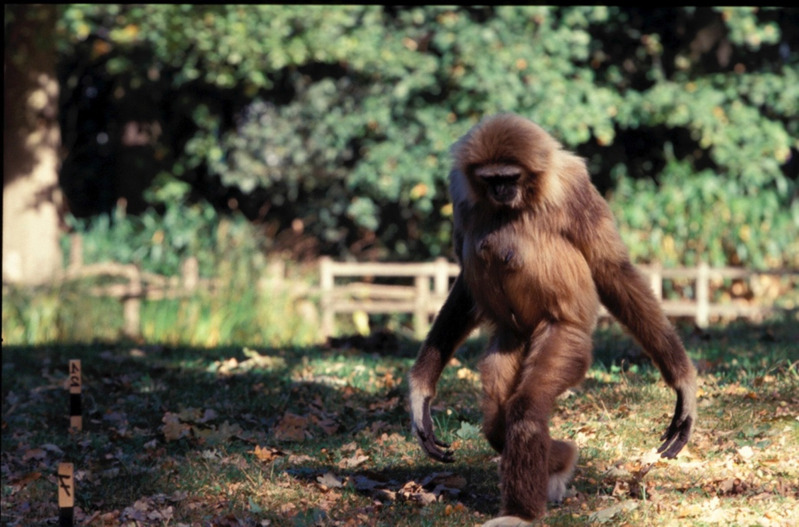
Bipedalism in *Hylobates*. Photograph courtesy of Evie Vereecke.

Yet, we also cannot ignore these data demonstrating that hylobatids – the only primates in our study with a long lumbar region *and* an orthograde body posture, essential characteristics for upright walking in humans (Williams & Russo, 2015) – practice bipedal locomotion more frequently and for longer distances than the other non-human primates (Figure 3). Our data are therefore consistent with a long-backed, orthograde LCA, which are foundational elements of the hylobatian hypothesis (Avis, 1962; Gregory, 1928; Keith, 1923; Morton, 1924; Tuttle, Butzer, & Blumenberg, 1974). We are not suggesting that the LCA was the size of modern hylobatids (see Grabowski & Jungers, 2017; Tuttle et al., 1974), nor was it likely to be a specialised brachiator, merely that it was orthograde (as all modern apes are) and may have lacked the short-stiff lumbar spine present in great apes. Importantly, several middle-to-late Miocene fossil hominoids such as *Oreopithecus* (Hammond et al., 2020), *Rudapithecus* (Ward et al., 2019), *Danuvius* (Böhme et al., 2019) and *Pierolapithecus* (Machnicki & Reno, 2020) are also reconstructed as long-backed, orthograde climbers with at least some capacity for arboreal bipedalism. Additionally, the earliest known hominin partial skeleton from 4.4 Ma *Ardipithecus ramidus* is hypothesised to possess an elongated lumbar region (Lovejoy et al., 2009).

These lines of evidence tentatively indicate that humans and hylobatids reflect the ancestral body form with respect to lumbar mobility and positional behaviour (for more similarities see Zichello, 2018). If true, then the short, stiff lumbar regions and correlated terrestrial knuckle-walking locomotion evolved in parallel in *Pan* and *Gorilla* as some have hypothesised (Böhme et al., 2019; Lovejoy et al., 2009; Lovejoy & McCollum, 2010; Machnicki & Reno, 2020; McCollum et al., 2010; Ward et al., 2019). This interpretation of our findings is subject to modification as more hominid fossils from the late Miocene of Africa and Eurasia are unearthed.

## Supplementary material

For supplementary material accompanying this paper visit https://doi.org/10.1017/ehs.2022.9. click here to view supplementary material

## Research transparency and reproducibility

All raw data used to formulate the ideas in this paper are available as supplemental documents.

## Supplemental information

Document S1, Excel file with a list of contributing AZA institutions and the primate sample size from each. Document S2, Sample survey distributed to primate caretakers at zoos. Movie S1, Female *Gorilla gorilla* moving bipedally at Zoo Atlanta.
