# Supplementary material for: Bipedal locomotion in zoo apes: Revisiting the hylobatian model for bipedal origins
Source: Evol Hum Sci. 2022 Mar 14;4:e12. doi: 10.1017/ehs.2022.9 (PMC10426021; doi:10.1017/ehs.2022.9)
Supplement: Supplementary file 1 [file S2513843X22000093sup001.zip › ZooSurvey PDF.pdf]

Observed Frequency of Bipedal Locomotion in Apes Under Human Care

Start of Block: Block 3

DARTMOUTH COLLEGE
Bipedal Locomotion in Apes Under Human Care

This project is being conducted by an undergraduate student with supervision by a faculty advisor from Dartmouth College, Hanover, NH, USA. It is a study of observed instances of bipedal locomotion in apes under human care. These instances should involve an ape moving a variable distance on two legs (walking). This study is not interested in instances where apes stand upright without moving on two legs.
Your participation is voluntary. Participation involves completion of a survey that should take roughly 6 minutes. You may choose to not answer any or all questions. If you are unable to complete it in one sitting, you may return to this survey at a later point in time. Your responses will be saved automatically and will be recorded after 1 week of inactivity.
The information collected will be completely confidential and anonymous. Questions about this project may be directed to:
Student: Kyle Rosen, Kyle.H.Rosen.20@dartmouth.edu
Faculty advisor: Dr. Jeremy DeSilva, Jeremy.M.Desilva@dartmouth.edu

---

If you agree to participate in this survey, click "I agree" below

○ I agree to participate (1)

○ I do not agree to participate (2)

End of Block: Block 3

Start of Block: Default Question Block

Name of your facility?

____

---

*

How many apes, in total, are in your collection regardless of species?

____

What species of ape do you provide care for? You may select multiple if applicable.

- ☐ Chimpanzee (Pan troglodytes) (1)
- ☐ Bonobo (Pan paniscus) (2)
- ☐ Gorilla (Gorilla s.p.) (3)
- ☐ Orangutan (Pongo s.p.) (4)
- ☐ Gibbon (Hylobates s.p.) (5)
- ☐ Siamang (Syndactylus s.p.) (6)

*

How many chimpanzees do you provide care for?

____

*

How many bonobos do you provide care for?

____

*

How many gorillas do you provide care for?

____

*

How many orangutans do you provide care for?

____

*

How many gibbons do you provide care for?

____

*

How many siamangs do you provide care for?

____

Have you made any behavioral observations of bipedal locomotion amongst any of your apes at any time?

- ○ Yes (1)
- ○ No (2)

End of Block: Default Question Block

Start of Block: Block 4

During this portion of the survey, we are interested in learning more about each individual ape that you provide care for. Please focus on one ape at a time when answering these short sets of questions. Please answer these questions for each ape regardless of whether or not they exhibit bipedal behavior. Once you finish answering these questions, the section will repeat as many times as apes you provide care for. If you have a large collection of apes and/or do not

have the time to comment on each one, please feel free to send this survey to your colleagues who can comment on the apes that you do not comment on.

---

*

How many apes can you comment on? Note: The number you answer here will determine how many times the following short section about individual apes repeats.

____

End of Block: Block 4

Start of Block: Ape Characteristics

What species of ape do your answers to the following questions pertain to?

- ○ Chimpanzee (Pan troglodytes) (1)
- ○ Bonobo (Pan paniscus) (2)
- ○ Gorilla (Gorilla s.p.) (3)
- ○ Orangutan (Pongo s.p.) (4)
- ○ Gibbon (Hylobates s.p.) (5)
- ○ Siamang (Syndactylus s.p.) (6)

Age

- ○ Adult (1)
- ○ Juvenile (2)
- ○ Infant (3)

Sex

- ◯ Male (1)
- ◯ Female (2)

Please select all that apply: I have observed bipedal locomotion in this ape at any point during....

- ☐ This past month (50)
- ☐ This past week (51)
- ☐ Today (52)
- ☐ No bipedal locomotion was observed in any of these timeframes (53)

How many times did this ape exhibit this behavior? Please give us your best numerical estimate if you do not recall the exact number of times. Please also indicate the appropriate timeframe (e.g. "X times per day/week/month").

____

On average, how many steps did this ape take in each instance?

- ◯ 1-3 (2)
- ◯ 3-5 (3)
- ◯ 5-7 (4)
- ◯ 7-9 (5)
- ◯ 10+ (6)

What were the circumstances for the observed instance(s) of bipedal locomotion? If there were multiple circumstances, please describe each one, such as, for example, if an ape moved bipedally while engaging in play behavior.

____

____

____

____

____

When you are ready, press "next" to fill out information on a different ape you provide care for.

- ◯ Next (1)

End of Block: Ape Characteristics

Start of Block: Final Block

Since the outbreak of COVID-19 and the subsequent removal of visitors from zoos, have you noticed a change in the frequency of bipedal locomotion in any of your apes? If so, in what way? Please make sure to specify which species if applicable.

____

____

Do you have any additional comments you would like to add regarding anything discussed in this survey?

End of Block: Final Block
